# NEOADJUVANT CHEMORADIOTHERAPY FOLLOWED BY TRANSHITAL ESOPHAGECTOMY IN LOCALLY ADVANCED ESOPHAGEAL SQUAMOUS CELL CARCINOMA: IMPACT OF PATHOLOGICAL COMPLETE RESPONSE

**DOI:** 10.1590/0102-672020210002e1621

**Published:** 2021-12-17

**Authors:** Iuri Pedreira Fillardi ALVES, Valdir TERCIOTI, João de Souza COELHO, José Antonio Possatto FERRER, José Barreto Campello CARVALHEIRA, Eduardo Baldon PEREIRA, Luiz Roberto LOPES, Nelson Adami ANDREOLLO

**Affiliations:** 1 Digestive Diseases Surgical Unit, Department of Surgery and Gastrocentro; 2 Clinical Oncology Division, Department of Internal Medicine; 3 Radiotherapy Division, University Hospital, School of Medical Sciences, State University Campinas - Unicamp, Campinas, Sao Paulo, Brazil

**Keywords:** Esophageal Neoplasms, Squamous Cell Carcinoma, Neoadjuvant Therapy, Surgical Oncology, Neoplasias Esofágicas, Carcinoma de Células Escamosas, Terapia Neoadjuvante, Oncologia Cirúrgica

## Abstract

**Background:**

Multimodal therapy with neoadjuvant chemoradiotherapy, followed by esophagectomy has offered better survival results, compared to isolated esophagectomy, in advanced esophageal cancer. In addition, patients who have a complete pathological response to neoadjuvant treatment presented greater overall survival and longer disease-free survival compared to those with incomplete response.

**Aim:**

To compare the results of overall survival and disease-free survival among patients with complete and incomplete response, submitted to neoadjuvant chemoradiotherapy, with two therapeutic regimens, followed by transhiatal esophagectomy.

**Methods:**

Retrospective study, approved by the Research Ethics Committee, analyzing the medical records of 56 patients with squamous cell carcinoma of the esophagus, divided into two groups, submitted to radiotherapy (5040 cGY) and chemotherapy (5-Fluorouracil + Cisplatin versus Paclitaxel + Carboplatin) neoadjuvants and subsequently to surgical treatment, in the period from 2005 to 2012, patients.

**Results:**

The groups did not differ significantly in terms of gender, race, age, postoperative complications, disease-free survival and overall survival. The 5-year survival rate of patients with incomplete and complete response was 18.92% and 42.10%, respectively (p> 0.05). However, patients who received Paclitaxel + Carboplatin, had better complete pathological responses to neoadjuvant, compared to 5-Fluorouracil + Cisplatin (47.37% versus 21.62% - p = 0.0473, p <0.05).

**Conclusions:**

There was no statistical difference in overall survival and disease-free survival for patients who had a complete pathological response to neoadjuvant. Patients submitted to the therapeutic regimen with Paclitaxel and Carboplastin, showed a significant difference with better complete pathological response and disease progression. New parameters are indicated to clarify the real value in survival, from the complete pathological response to neoadjuvant, in esophageal cancer.

## RESUMO

**Racional:**

A terapia multimodal com quimioradioterapia neoadjuvantes, seguido de esofagectomia tem oferecido melhores resultados de sobrevida, em comparação à esofagectomia isolada, no câncer do esôfago avançado. Além disso, os doentes que apresentam resposta patológica completa ao tratamento neoadjuvante, têm evoluido com maior sobrevida global e maior sobrevida livre de doença em comparação aos que apresentam resposta incompleta.

**Objetivo:**

Comparar os resultados de sobrevida global e sobrevida livre de doença entre os doentes com resposta completa e incompleta, submetidos à quimioradioterapia neoadjuvante, com dois esquemas terapêuticos, seguidos de esofagectomia transhiatal.

**Métodos:**

Estudo retrospectivo, aprovado pelo Comitê de Ética em pesquisa, analisando os prontuários de 56 doentes, divididos em dois grupos de pacientes, submetidos a radioterapia (4400 a 5400 cGY) e quimioterapia (5-Fluorouracil+Cisplatina *versus* Paclitaxel+Carboplatina) neoadjuvantes e posteriormente a tratamento cirúrgico, no período de 2005 a 2012, portadores de carcinoma espinocelular do esôfago.

**Resultados:**

Os grupos não diferiram significativamente quanto ao gênero, raça, idade, complicações pós-operatórias, sobrevida livre de doença e sobrevida global. A sobrevida em 5 anos de doentes com resposta incompleta e completa foram, respectivamente, 18,92% e 42,10% (p>0,05). Entretanto, os doentes que receberam Paclitaxel+Carboplatina, tiveram melhores respostas patológicas completas à neoadjuvância, em comparação ao 5-Fluorouracil+Cisplatina (47,37% *versus* 21,62% - p=0,0473, p<0,05).

**Conclusões:**

Não houve diferença estatística na sobrevida global e na sobrevida livre de doença dos doentes que apresentaram resposta patológica completa à neoadjuvância. Os doentes submetidos ao esquema terapêutico com Paclitaxel e Carboplastina, mostraram diferença significativa com melhor resposta patológica completa e evolução da doença. Novos parâmetros são indicados para esclarecer o real valor na sobrevida, da resposta patológica completa à neoadjuvância, no câncer de esôfago.

## INTRODUCTION

 The esophageal cancer treatment has progressed in the last fifteen years. The low cure rate with the exclusive surgical treatment, stimulated the inclusion of multimodal treatments. In the past, radiotherapy was used as the only form of treatment for squamous cell carcinoma, demonstrating in some studies, results similar to those of surgery. Recently, chemotherapy and associated radiotherapy have shown better survival results, while radiotherapy as the definitive modality has been reserved for patients who cannot receive chemotherapy ^1^ ^,^ ^2^ ^,^ ^3^ ^,^ ^9^ ^,^ ^10^ ^,^ ^13^ ^,^ ^14^ ^,^ ^22^ ^,^ ^23^.

 Several clinical trials and meta-analyses have shown the best survival of patients undergoing neoadjuvant chemoradiotherapy ^1^ ^,^ ^6^ ^,^ ^10^ ^,^ ^11^ ^,^ ^28^ ^,^ ^31^. The inclusion of systemic chemotherapy in the treatment regimens with multimodality, to control distant micrometastatic disease and improve the effects of local radiation through its radiosensitizing effect, made this practice routine, with the main objective of achieving cytoreduction and decreased staging (downstage) ^16^ ^,^ ^22^. Another advantage of neoadjuvant is the fact that cytoreduction and the consequent tumor reduction, the patient eats better, gains weight and acquires a more appropriate nutritional status for a possible surgical procedure, in addition to improving the quality of life due to the lower rate of dysphagia ^1^ ^,^ ^3^ ^,^ ^24^ ^,^ ^26^. The several advantages, such as those mentioned above, are notorious, especially when compared to other types of treatments carried out together or isolated. There is a higher rate of R0 resections, in addition to the possibility of a complete response to neoadjuvant therapy, that is, a complete absence of tumor cells in the surgical specimen ^4^ ^,^ ^5^ ^,^ ^11^ ^,^ ^12^ ^,^ ^13^ ^,^ ^15^ ^,^ ^20^ ^,^ ^21^ ^,^ ^27^.

 Therefore, neoadjuvant chemoradiotherapy has been widely used in many Oncology Services around the world. However, in clinical practice, these good results have not always meant better overall and disease-free survival, with controversies about the real benefits of neoadjuvant in the treatment of squamous cell carcinoma of the esophagus ^6^ ^,^ ^12^ ^,^ ^21^ ^,^ ^23^ ^,^ ^26^ ^,^ ^31^.

 The aim of this study was to analyse the results of global and disease-free survival, according to the complete or incomplete response to neoadjuvant treatment, followed by transhiatal esophagectomy in squamous cell carcinoma of the esophagus, at the Unicamp University Hospital, from 2005 to 2012, as well as their clinical characteristics

## METHODS

 The medical records of patients submitted to transhiatal esophagectomy, by the same team of surgeons, from 2005 to 2012, were reviwed. This period included the beginning of trimodal treatment (neoadjuvant with chemoradiotherapy followed by surgery), for advanced squamous cell carcinoma of the esophagus. The research was approved by the Unicamp Research Ethics Committee (nº 1.612.155).

### 1. INCLUSION CRITERIA

The inclusion criteria were:

I) Patients with esophageal neoplasia submitted to transhiatal esophagectomy at Unicamp University Hospital;
II) Histopathological findings showing squamous cell carcinoma;
III) Tumor located in the middle and lower thirds of the esophagus;
IV) Patients submitted to chemoradiotherapy neoadjuvant.

### 2. EXCLUSION CRITERIA

The exclusion criteria were:

I) Patients with medical records not found or with incomplete data;
II) Patients who underwent surgical or neoadjuvant treatment in another service;
III) Patients who had only neoadjuvant chemotherapy or only radiotherapy;
IV) Histopathological findings of adenocarcinoma.

### 3. STUDY VARIABLES

 The variables collected for the study were: age, gender, race, the tumor site, staging, histopathological type, neoadjuvant treatment modalities, response to neoadjuvant, postoperative complications and follow-up (disease-free time) and 5-year survival).

### 4. PATIENTS

 In the evaluation period (2005 to 2012) 63 patients were operated on, 7 of whom were excluded, according to the exclusion criteria. The final sample of the study was 56 (N) patients who underwent neoadjuvant with 2 therapeutic regimens and then underwent transhiatal esophagectomy.

### 5. NEOADJUVANT TREATMENT

 The radiotherapy and chemotherapy treatments were performed, respectively, at the Radiotherapy and Clinical Oncology Division of the Unicamp University Hospital. The standard total radiation dose was 5040 cGY, divided into 25 to 30 sessions of 180 cGY. 

 Chemotherapy treatment employed two therapeutic regimens:

a) two cycles of cisplatin, the second cycle being administered 21 days after the first cycle (75 mg / m2 between D1 or D4) associated with 5-Fluorouracil (5-FU) (1000 mg / m2 in continuous infusion in D1 to D5 ) ^2^ ^,^ ^4^ ^,^ ^14^.
b) Paclitaxel together with Carboplatin, both medications being administered on days 1, 8, 15, 22 and 29. The dose of Paclitaxel was 50mg / m2 and the dose of Carboplatin was calculated with the absolute dose, multiplying the body area below the target curve by the patient's glomerular filtration rate added to 25 ^14^ ^,^ ^29^ ^,^ ^30^.

### 6. SURGICAL TREATMENT

 All patients were submitted to surgical treatment by the same team, with the same standardization, being carried out between 30 and 60 days after the end of neoadjuvant. The surgical technique used was transhiatal subtotal esophagectomy, using median laparotomy and associated left lateral cervicotomy. Reconstruction of gastrintestinal transit was performed by making the isoperistaltic gastric tube followed by cervical esophagogastric anastomosis. All patients underwent pyloromyotomy, and jejunostomy for early postoperative enteral nutritional support ^3^ ^,^ ^25^.

### 7. THERAPEUTIC RESPONSE

 The tumor response to neoadjuvant therapy was assessed with the anatomopathological study of the surgical specimen, establishing two possible findings:

a) complete response to neoadjuvant treatment, considered as responders, when tumor cells were not found in the histopathological studies of the surgical specimen.
b) incomplete response to neoadjuvant treatment, considered non-responders, when neoplasia or residual foci of tumor cells were found in the histopathological studies of the surgical specimen.

### 8. STAGING

 Tumor staging was based on the pathological findings of the surgical specimen, according to the TNM classification criteria for esophageal squamous cell carcinoma, recommended by UICC ^18^.

### 9. STATISTICAL ANALYSIS

 The age of the patients calculated and considered for the description of the studied specimens was the age of the patient on the date of the surgery (the date of the surgery subtracted from the date of the patient's birth). The information was collected in 2018.

 Patients' survival was calculated by subtracting the date of death from the date of surgery. The sample profile was described according to the variables under study, in frequency Tables of categorical variables with absolute (n) and percentage (%) frequency values, and descriptive statistics for numerical variables, with mean values, standard deviation, minimum values, maximum and median.

 The Chi-square test was used to compare categorical variables (χ2) and, when necessary, Fisher's exact test. The Mann-Whitney test was used to compare numerical variables. COX regression analysis was employed to assess survival in relation to response to treatment and choice of chemotherapeutic agents. The level of significance adopted for the study was 5% (p < 0.05).

 For statistical analysis, the following computer programs were used: The SAS System for Windows (*Statistical Analysis System*), version 9.4. SAS *Institute Inc,* 2002-2008, Cary, NC, USA ^8^.

## RESULTS

 Among the 56 patients studied, 80.36% (N = 45) were white and 19.64% (N = 11) were brown. The distribution by gender was 48 males (85.71%) and 8 females (14.29%). The mean age of patients was 55.23 years, with a median of 54 years, standard deviation of 8.12 years, and a minimum age of 40 years and a maximum of 68 years. There was no statistical difference between the two groups. The tumors were located in the middle third in 36 cases (64.29%) and in the distal third in 20 cases (35.71%).

 Postoperative complications are shown in Table 1.

**Table 1:** Prevalence of postoperative complications among patients with complete and incomplete response to neoadjuvant treatment and statistical analysis

Complications (%)		Incomplete Response (%)	Complete Response (%) (p)
Bleeding (chest drain)	No	35 (94,59)	18 (94,74)
N=3 (5,36%)	Yes	2 (5,41)	1 (5,26) (p=1,0000 p>0,05)
Anastomosis fistula	No	29 (78,38)	14 (73,68) (p=0,7449 p>0,05)
N=13 (23,21%)	Yes	8 (21,62)	5 (26,32)
Anastomosis stenosis	No	26 (70,27)	15 (78,95)
N=15 (26,79%)	Yes	11 (29,73)	4 (21,05) (p=0,4875 p>0,05)
Chest drainage	No	7 (18,92)	4 (21,05)
N=45 (80,36%)	Yes	30 (81,08)	15 (78,95) (p=1,0000 p>0,05)
Bronchopneumonia	No	22 (59,46)	13 (68,42%)
N=21 (37,50%)	Yes	15 (40,54)	6 (31,58) (p=0,5119 p>0,05)
Urinary infection	No	37 (100)	18 (94,74)
N=1 (1,79%)	Yes	-	1 (5,26)
Cardiac complications	No	35 (94,59)	19 (100)
N=2 (3,57%)	Yes	2 (5,41)	- (p=0,5435 p>0,05)
Perioperative deaths	No	34 (91,89)	16 (84,21)
N=6 (10,71%)	Yes	3 (8,11)	3 (15,79) (p=0,3971 p>0,05)

 There were no significant differences for postoperative complications and perioperative deaths between the two groups.

 The etiologies of the deaths were bronchopleuropulmonary complications in 5 cases and abdominal sepsis in one case.

 The histopathological findings of the surgical specimens were studied and presented according to the TNM, degree of differentiation and staging by UICC / AJCC / WHO ^18^ (Tables 2 and 3).

**Table 2:** T, N, M staging and differentiation grades in surgical specimens (M = 0 in preoperative clinical staging).

Staging		Frequences (%)
T	0	21 (38,89)
	2	14 (25,93)
	3	19 (35,19)
N	0	35 (62,50)
	1	10 (17,86)
	2	9 (16,07)
	3	2 (3,57)
M	0	56 (100)
	1	0 -
Differentiation grades	1	2 (3,57)
	2	43 (76,79)
	3	11 (19,64)

**Table 3:** Stage of the patients according to histopathological analysis of the surgical specimens (M = 0 in the preoperative clinical staging).

Stage	Frequences (%)
0	19 (33,93)
IA	2 (3,57)
IB	5 (8,93)
IIA	9 (16,07)
IIB	6 (10,71)
IIIA	9 (16,07)
IIIB	4 (7,14)
IIIC	2 (3,57)

 During the outpatient postoperative follow-up period, tumor recurrence was recorded in 20 cases (35.71%), and the remaining 36 cases (64.29%) did not present recurrence. Tumor recurrences were observed in several places, in some cases, in more than one organ. The recurrences diagnosed, during the period of analysis, were: lungs (5 cases), gastric tube (5 cases), cervical lymph nodes (4 cases), liver (2 cases), bones (2 cases) and cerebral (2 cases).

 The higher percentage of recurrence was found among patients with incomplete response (43.24%) compared with patients with complete response (21.05%), however, without statistical difference (Table 4).

**Table 4:** Tumor relapse among patients with complete and incomplete response

Tumor relapse	Incomplete response (%)	Complete response (%) (p)
No	21 (56,76)	15 (78,95)
Yes	16 (43,24)	4 (21,05) (p=0,1008 p>0,05)

 Two neoadjuvant therapeutic schemes, associated to radiotherapy, were employed: Cisplatin + 5-Fluorouracil in 39 patients (69.64%) and Paclitaxel + Carboplatin in the remaining 17 patients (30.36%). The use of one or the other scheme depended on the temporal evolution of these treatments, according to published randomized studies ^1^ ^,^ ^2^ ^,^ ^14^ ^,^ ^21^ ^,^ ^28^ ^,^ ^29^ ^,^ ^30^.

 The results of the surgical specimens analysed, according to the different neoadjuvants schemes, are showed in Table 5, with a statistically significant difference for the Paclitaxel + Carboplatin group (p <0.05).

**Table 5:** The chemotherapy neoadjuvant employed and the complete and incomplete pathological response

Neoadjuvancy	Incomplete (%)	Complete (%) (p)
Cisplatin + 5-Fluorouracil	29 (78,38)	10 (52,63)
Paclitaxel + Carboplatin	8 (21,62)	9 (47,37) (p=0,0473 p<0,05)

**Figure 1:**
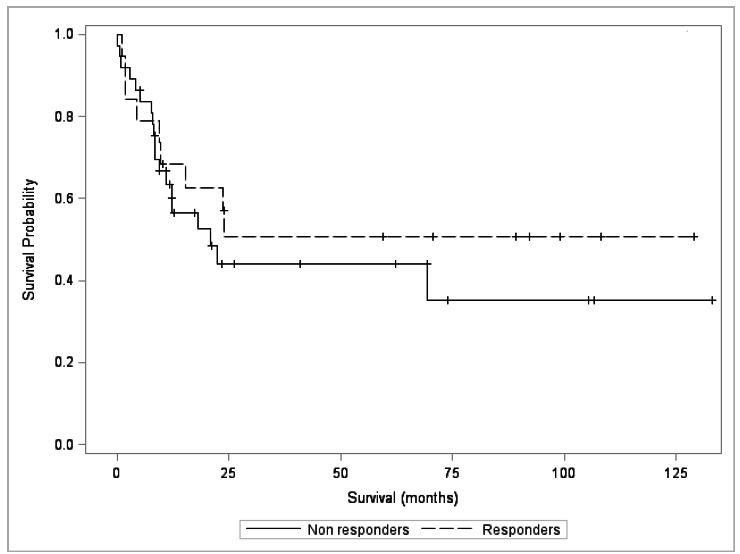
Kaplan-Meier survival curve for groups with complete response (responders) and incomplete response (non responders)

Seven patients (18.92%) with incomplete response and eight patients (42.10%) with complete response, had a survival greater than 5 years, with no statistical difference (p = 0.4614 p> 0.05), as shown in Table 6 and the Kaplan-Meier curve in Figure 1.

**Table 6:** The 5-year survival rate, compared with the neoadjuvant chemotherapy scheme employed.

Survival	Cisplatin + 5-Fluorouracil (%)	Paclitaxel + Carboplatin (%)
< 5 anos	29 (74,36)	12 (70,59)
> 5 anos	10 (25,64)	5 (29,41)

**Figure 2:**
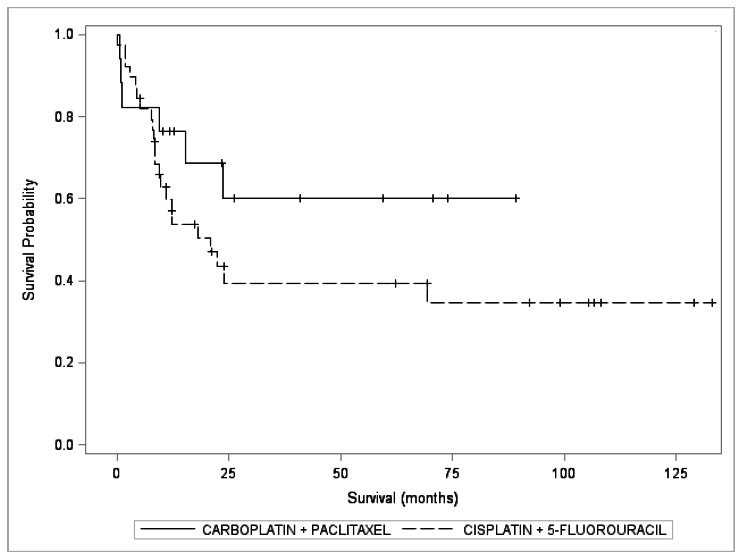
Kaplan-Meier survival curve for the groups according to the neoadjuvant chemotherapy schemes employed

 Comparing the two neoadjuvants schemes and the 5-year survival, there was no statistical difference (p = 0.1918, p> 0.05), as shown in Table 6 and the Kaplan-Meier curve Figure 2:

## DISCUSSION

 The purpose of neoadjuvant treatment is to obtain the complete pathological response (pCR). This study showed a pCR of 33.93%, which is in according with the literature ^20^ ^,^ ^24^. But, differently from what would be expected, this response was not reflected in survival.

 The clinical staging of advanced tumors (Table 3), associated with a large number of patients with incomplete response (non-responders) to neoadjuvant treatment, may be the reason that better survival was not recorded for patients with complete response (responders). In addition, these results may be related to high rates of recurrence, directly influencing disease-free survival.

 The main recurrence places observed in this study were far from the treated organ, and in lower percentages than other studies ^10^ ^,^ ^14^ ^,^ ^27^. This fact may mean a possible presence of metastatic and undetected disease at the time of surgery, compromising the real value of the complete pathological response (pCR), since in this finding there was no statistical difference between the groups analyzed.

 These discussions corroborate the fact that, perhaps, the available imaging exams to assess the clinical staging are currently limited in accuracy, and do not show the real dimension of the extension of the disease ^11^ ^,^ ^15^ ^,^ ^21^ ^,^ ^24^. Therefore, safely assessing the reduction in staging, just comparing the postneoadjuvant pathological staging with the preneoadjuvant clinical staging, may be controversial. Unfortunately, there are no better tools currently available for preoperative staging. When better methods become available, it will be possible to more accurately identify downstaging ^7^. Even the best techniques available at the moment, such as endoscopic ultrasound and PET-CT, which offer better specificity and sensitivity for preoperative staging, they are not used routinely.

 Patients who have a complete response to neoadjuvant therapy are expected to have greater overall survival, as recorded in the present study, reflecting a longer 5-year survival in the group of complete responses (42.10%) *versus* incomplete responses (18.92%), however, with no statistically significant difference.

 Previous studies carried out at the same Service analyzing 177 patients with squamous cell carcinoma undergoing neoadjuvant therapy followed by transhiatal esophagectomy, from 1983 to 2014, were analyzed, 34 cases with complete pathological response (19.2%). Among the 34 cases, 9 had been submitted to radiotherapy and 25 to chemoradiotherapy (non-individualized Cisplatin + 5-FU and Paclitaxel + Carboplatin regimens). Comparing the survival curves of the two groups, there was no statistically significant difference between the two groups (p> 0.05). However, patients undergoing chemoradiotherapy had a longer survival time after 60 months of follow-up (52% *versus* 23%) ^4^.

 Rizzetto et al. ^19^, in 2008 analysing the difference between *en bloc* esophagectomy and transhiatal esophagectomy, performed a review of medical records from 1992 to 2005, in patients with esophageal neoplasia who underwent neoadjuvant followed by surgery. A total of 58 patients underwent *en bloc* esophagectomy and 18 to transhiatal esophagectomy were compared. The complete pathological response occurred in 17 (29.3%) of the 58 patients. The median follow-up was 34.1 months after *en bloc* resection and 18.3 months after transhiatal resection (p = 0.18, p> 0.05). Overall survival at 5 years and survival in patients with residual disease after neoadjuvant therapy were significantly better with a *en bloc* resection (overall survival: 51% for *en bloc* resection and 22% for transhiatal resection (p = 0.04, p < 0.05); Survival with residual disease: 48% for *en bloc* resection and 9% for transhiatal resection (p = 0.02, p <0.05). Survival in patients with complete pathological response tended to be better after *en bloc* resection (*en bloc*, 70%; transhiatal, 43%; p = 0.3, 0> 0.05). The authors concluded that *en bloc* resection provides a survival advantage for patients after neoadjuvant therapy in compared to a transhiatal resection, particularly for those with residual disease.

 The best complete response rate occurred in the group that used the neoadjuvant chemotherapy regimen with Paclitaxel + Carboplatin compared to the Cisplatin + 5FU regimen, with a statistically significant difference (p = 0.0473, p <0.05), despite a small number of patients in the first group. In future studies, with a greater number of patients treated with the current neoadjuvant protocol, patients with a complete response may have better survival. 

 van Meerten et al. ^29^, in 2006, studied the efficacy and safety of preoperative chemoradiotherapy composed of Paclitaxel + Carboplatin and concomitant radiotherapy for patients with resectable esophageal cancer (T2-3N0-1M0). The treatment consisted of Paclitaxel 50 mg / m2 and AUC = 2 of Carboplatin on days 1, 8, 15, 22 and 29 and concomitant radiotherapy (41.4 cGY in 23 fractions, 5 days a week), followed by esophagectomy. All 54 patients who entered the regimen completed chemoradiatherapy. The 3-4 toxicities grades were: neutropenia 15%, thrombocytopenia 2% and esophagitis 7.5%. After the final of chemoradiotherapy, 63% had an endoscopic response. Fifty-two patients (96%) underwent resection. The postoperative mortality rate was 7.7%. All patients had R0 resection. The percentage of complete pathological response was 25%, with 36.5% having less than 10% of residual tumor cells. The mean follow-up time was 23.2 months and the disease-free survival after 30 months was 60%. The authors concluded that the weekly Paclitaxel and Carboplatin associated to radiotherapy proved to be a very tolerable regimen and can be administered on an ambulatory outpatient, causing gradual tumor reduction and allowing radical resections in almost all patients.

 van de Schoot et al. ^30^, in 2007, carried out a phase II study to assess the viability and efficacy of a neoadjuvant chemoradiotherapy based on Paclitaxel and Carboplatin followed by surgery in patients with stage II-III esophageal cancer. From January 2002 to November 2004, 50 potentially resectable patients with stage II-III esophageal cancer received chemotherapy with Paclitaxel, Carboplatin and 5-FU in combination with 45 cGY radiotherapy in 25 fractions. Surgery was indicated between 6 to 8 weeks after the completed neoadjuvant treatment. Toxicity was mild and 84% of patients completed the entire proposed protocol. Forty-seven patients (94%) were operated with curative intent (transhiatal esophagectomy n = 44 cases, transthoracic esophagectomy n = 3 cases). The complete pathological response was achieved in 18 operated cases (38%). R0 resection was achieved in 45 cases (96%). Postoperative complications were comparable with other studies and four postoperative deaths (4.5%) were recorded. After an average follow-up of 41.5 months (from 21 to 59 months), the estimated survival at 3 and 5 years was 56% and 48%, respectively. The estimated three-year survival in responders was 61% and in non-responders was 33%. The authors concluded that neoadjuvant chemoradiotherapy for the treatment of patients with stage II-III esophageal cancer was feasible. The results were encouraging, with a high complete pathological regression of the tumor and a R0 resection rate and an acceptable morbidity and mortality.

 Takeda et al. ^23^, in 2019, analysed the tumor regression grade after trimodal therapy (neoadjuvant chemoradiotherapy followed by surgery) in 134 patients, both in patients with squamous cell carcinoma (90 cases) and in adenocarcinoma (34 cases) using the Ryan score, used in rectal tumors. They conclude that there was a significant correlation with the histological type, clinical and pathological stage, in the mean follow-up of 31.1 months. The study employed multivariate analysis which showed that Ryan's score can safely predict survival and systemic and lymphatic recurrence. The same authors reflecting on this important issue in the treatment of esophageal cancer, conclude that future studies should evaluate different neoadjuvant chemotherapy schemes, as well as irradiation in different fields, in addition to studies comparing neoadjuvant therapies with definitive chemoradiotherapy.

 Figueroa-Giralt et al. ^13^, in 2020, proposed the use of the lymphoparietal index in the survival of esophageal cancer, after analyzing the treatment of 58 patients, treated with neoadjuvant or adjuvant therapy according to tumor stage and esophagectomy by minimally invasive techniques and cervical esophageal-gastric anastomosis. They conclude that the main independent prognostic factors for survival of more than three years, in a Latin American country, are: gender, anterior mediastinal traction, anastomotic fistula, classification N, TNM stage and lymphoparietal index.

 Takeda et al. ^25^, in 2019, published their experience with transhiatal esophagectomy in 149 patients and concluded that this access route is a good option, it is associated with reduced perioperative morbidity, shorter hospital stay and decreased in-hospital mortality, especially in cases of transhiatal laparoscopic esophagectomy. In addition, with proper patient selection, transhiatal esophagectomy can preserve the quality of lymphadenectomy of positive lymph nodes.

 The present research, as it is a retrospective and non-randomized study, has limitations. However, since then, with the finding that the neoadjuvant regimen with Paclitaxel + Carboplatin has shown to have a better pathological response rate, this has been the protocol used in the Service, meaning that in the future, the casuistic will be more expressive and may add a better survival rate disease-free and 5-year survival. In addition, transhiatal esophagectomy showed acceptable rates of postoperative complications and in accordance with the literature ^17^ ^,^ ^25^, therefore, still indicated in selected cases.

 Finally, considering the results obtained in this research, the authors emphasize the importance of further research to clarify the real value of chemoradiotherapy in the complete pathological response in esophageal cancer, for survival rates.

## CONCLUSIONS

 The results obtained in the present research, permit the following conclusions:

1. The analysis of overall survival and disease-free survival showed no statistically significant difference between patients who had a complete and incomplete pathological response, who underwent neoadjuvant chemoradiotherapy followed by transhiatal esophagectomy, during the follow-up period of this research.
2. A better and statistically significant complete pathological response (responders) was observed for the group of patients undergoing the therapeutic scheme with Paclitaxel and Carboplatin, compared to the group that used Cisplatin and 5-Fluorouracil. However, there was no statistical difference in the overall survival rate between these two therapeutic options.
